# A novel physiological role for cardiac myoglobin in lipid metabolism

**DOI:** 10.1038/srep43219

**Published:** 2017-02-23

**Authors:** Ulrike B. Hendgen-Cotta, Sonja Esfeld, Cristina Coman, Robert Ahrends, Ludger Klein-Hitpass, Ulrich Flögel, Tienush Rassaf, Matthias Totzeck

**Affiliations:** 1 University Hospital Essen, Medical Faculty, West German Heart and Vascular Center, Department of Cardiology and Department of Angiology, Hufelandstr. 55, 45147 Essen, Germany; 2 Leibniz-Institut für Analytische Wissenschaften–ISAS e.V. Otto-Hahn-Str. 6b, 44227 Dortmund, Germany; 3 University Hospital Essen, Institute of Cell Biology, Medical Faculty, Virchowstr. 173, 45122 Essen, Germany; 4 University Hospital Düsseldorf, Department of Molecular Cardiology, Universitätsstr. 1, 40225 Düsseldorf, Germany

## Abstract

Continuous contractile activity of the heart is essential and the required energy is mostly provided by fatty acid (FA) oxidation. Myocardial lipid accumulation can lead to pathological responses, however the underlying mechanisms remain elusive. The role of myoglobin in dioxygen binding in cardiomyocytes and oxidative skeletal muscle has widely been appreciated. Our recent work established myoglobin as a protector of cardiac function in hypoxia and disease states. We here unravel a novel role of cardiac myoglobin in governing FA metabolism to ensure the physiological energy production through *β*-oxidation, preventing myocardial lipid accumulation and preserving cardiac functions. *In vivo*^1^H magnetic resonance spectroscopy unveils a 3-fold higher deposition of lipids in mouse hearts lacking myoglobin, which was associated with depressed cardiac function compared to wild-type hearts as assessed by echocardiography. Mass spectrometry reveals a marked increase in tissue triglycerides with preferential incorporation of palmitic and oleic acids. Phospholipid levels as well as the metabolome, transcriptome and proteome related to FA metabolism tend to be unaffected by myoglobin ablation. Our results reveal a physiological role of myoglobin in FA metabolism with the lipid accumulation-suppressing effects of myoglobin preventing cardiac lipotoxicity.

Myoglobin, a member of the heme globin family, is a multifunctional protein playing a critical role in biological processes, protecting the cardiovascular system^1^. Beyond the dioxygen (O_2_) buffering capacity, the activity of myoglobin targets at nitric oxide (NO) binding, catalyzation of NO dioxygenation, and nitrite reduction^2^,^3^,^4^,^5^,^6^,^7^. Taking advantage of the myoglobin knockout (*Mb*^−/−^) mouse we identified myoglobin as an O_2_ sensor in cardiomyocytes as well as in smooth muscle cells^5^,^8^,^9^. Deoxygenated myoglobin protects the heart from short phases of hypoxia and from myocardial ischemia/reperfusion injury *via* reduction of nitrite to NO and modulation of mitochondrial function^5^,^10^. Vascular myoglobin furthermore plays a critical role in regulating hypoxic vasodilation and controlling blood pressure^11^.

Fatty acids (FAs) are the main energy source of the heart. They derive from triglycerides in the blood and provide the majority of cofactors necessary for mitochondrial oxidative phosphorylation^12^. FAs are not stored intracellularly over a long-term period, but are immediately oxidized for ATP generation^13^,^14^. Only a few triglyceride stores serve as temporary endogenous source of FAs and exhibit a dynamic nature with a short mean turnover time^12^,^14^,^15^. This fine tuned FA metabolism by a balanced uptake and oxidation is absolutely necessary for covering cardiac energy requirement, preserving normal heart function and preventing lipid accumulation. The precise underlying mechanisms of the complex control of FA metabolism are not completely solved. Recent experimental and clinical studies using ^1^H magnetic resonance spectroscopy (^1^H MRS) indicated that perturbation of this homeostasis leads to cardiac dysfunction caused or aggravated by myocardial lipid deposition, a process termed cardiac lipotoxicity^16^,^17^,^18^,^19^. These studies provided evidence that failure of intracellular triglyceride-derived FAs mobilization induces triglyceride accumulation. Whether the myocardial triglyceride overload arises by impaired regulation of FA binding and channeling to particular metabolic fate, respectively, still awaits clarification.

Considering the cardioprotective role of myoglobin, a given affinity to FAs^20^,^21^,^22^,^23^,^24^ and an observed cardiac preference of glucose utilization in the absence of myoglobin^14^ it is tempting to speculate about a critical role of myoglobin in FA metabolism. To disclose a relevant functional role of myoglobin in preventing FA accumulation, we assessed lipid deposition and composition, metabolome alterations and cardiac function as well as the binding properties of myoglobin to FAs. Furthermore, we analyzed the regulation of opportunistic substitutes on the transcriptional and translational level in *Mb*^−/−^ mice. We provide evidence that ablation of myoglobin provokes a minor utilization of FA resulting in 3-fold higher deposition of triglycerides, which incorporate mostly palmitic and oleic acids in the heart. Oxygenated as well as metmyoglobin bind to these FAs - with a higher affinity to oleic acid. Our results also demonstrate that neither the metabolome nor the transcriptome nor proteome related to the FA metabolism tend to be altered by myoglobin deletion. Importantly, the lipid accumulation due to the lack of myoglobin entails depressed cardiac functions and heart atrophy. These findings point to a physiological role of myoglobin in FA metabolism.

## Results

### Myoglobin deletion induces lipid accumulation in the myocardium

**Figure 1:**
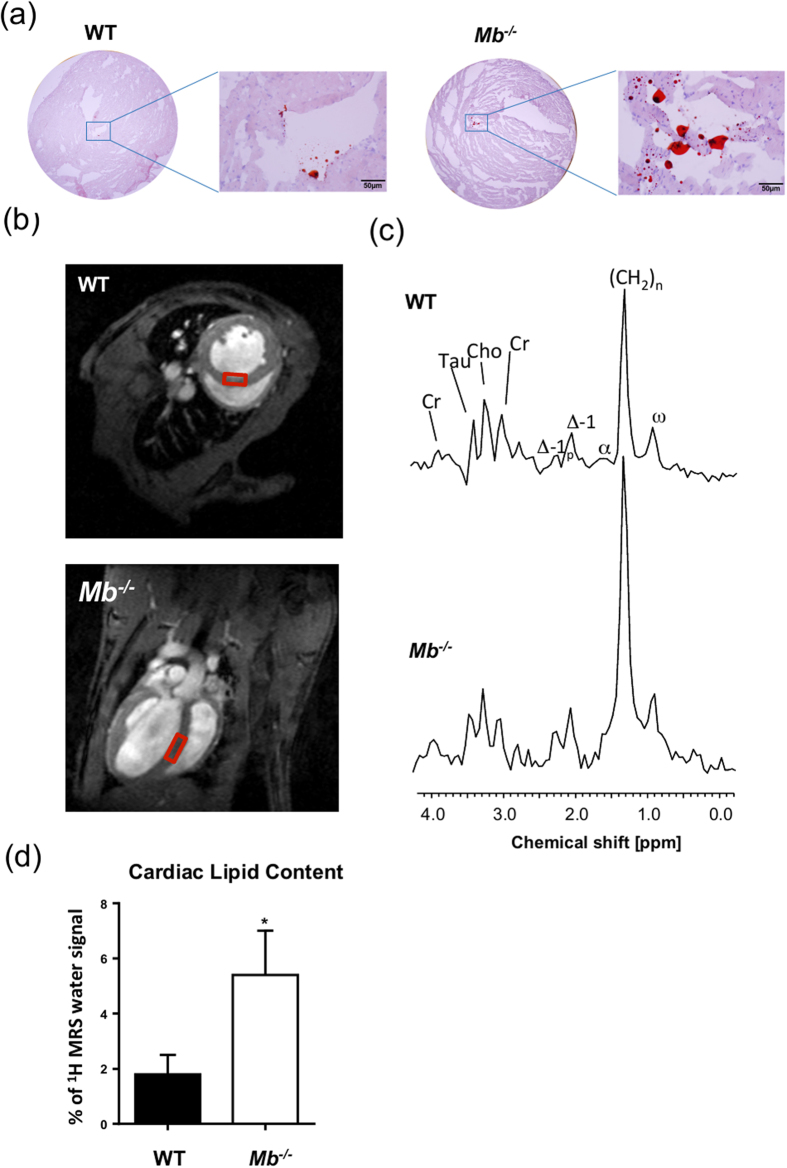
Myoglobin ablation provokes cardiac lipid accumulation. (**a**) Representative images of wild-type (WT) and myoglobin-deficient (*Mb*^−/−^) mouse heart slices stained with Oil Red O (bars represent 50 μm). (**b**) Short (top) and long (bottom) axis slices indicating the localization of the voxel used for spectroscopy in the interventricular septum. Spectra were gated to cardiac and respiratory motion and acquired with water suppression. (**c**) Characteristic volume-selective ^1^H MR spectra from hearts of a WT (top) and *Mb*^−/−^ (bottom) mouse indicating strongly increased lipid levels in the moycardium of the mutant. Assignment of proton signals: Cr, creatine; Tau, taurine; Cho, choline; Δ-1_p_, next to polyunsatured carbons; Δ-1, next to monounsatured carbons; β, bound to the β-carbon of FA; (CH_2_)_n_, methylene groups of FA; ω, terminal methyl group of FA. (**d**) Quantitative analysis of the lipid content in WT and *Mb*^−/−^ mouse hearts (values are mean ± SD; *P* < 0.05; *n* = 5).

Sustained alterations in cardiac substrate utilization for ATP production toward glucose is accompanied in large part by lipid deposition in the heart^16^,^17^,^18^,^19^,^25^. Considering that hearts lacking myoglobin exhibit a diminished *β*-oxidation of FA in favor of metabolizing glucose^6^ the cardiac tissue should contain more lipids. To estimate differences between 8-month-old *Mb*^−/−^ and wild-type (WT) mouse hearts we first used the oil-red-O staining of sections from frozen tissue in order to visualize cardiac neutral lipid contents. As expected, hearts lacking myoglobin exhibit a higher level of lipid accumulation in comparison to WT mouse hearts (Fig. 1a). To further quantify the lipid content we performed localized non-invasive ^1^H MRS in 8-month-old *Mb*^−/−^ and WT mouse hearts. As indicated in end-diastolic short and long axis slices in Fig. 1b, the spectroscopic voxel (6 μl) was placed in the interventricular septum to avoid signal contaminations from pericardial fat. Representative water-suppressed ^1^H MR spectra are illustrated in Fig. 1c for each genotype, where resonances for creatine, taurine, choline and several signals originating from lipids (see Figure legend for more details) can be unequivocally resolved. While signals for the first metabolites were comparable between the groups, all lipid peaks were clearly increased in hearts lacking myoglobin. Quantification of the spectra revealed that *Mb*^−/−^ hearts are characterized by a threefold higher lipid content compared to WT mouse hearts (5.5% ± 1.5% *vs*. 1.8% ± 0.7% of ^1^H MRS water signal, p = 0.034; Fig. 1c,d). This disparity was not apparent in 3-month old *Mb*^−/−^ and WT mice pointing to a delayed response to genetic ablation of myoglobin over time potentially due to failing compensatory mechanisms (2.2% ± 0.6% *vs*. 2.0% ± 0.5% of ^1^H MRS water signal, p = 0.8; see Supplementary Fig. S1).

**Figure 2:**
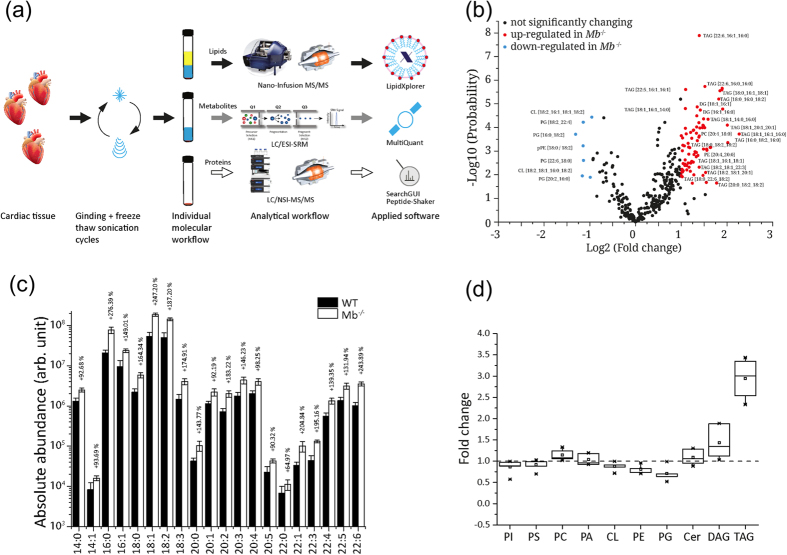
Myoglobin deletion entails formation of triglycerides. (**a**) SIMPLEX (**SI**multaneous **M**etabolite, **P**rotein, **L**ipid **EX**traction procedure) workflow. (**b**) Volcano plot of 363 quantified lipids. Red dots mark significantly up- and light blue dots significantly down-regulated lipids in myoglobin-deficient (*Mb*^−/−^) mouse hearts compared to wild-type (WT) mouse hearts. Black dots reflect no significantly changes (n = 3). (**c**) Bar graph of absolute abundance of different triglycerides in WT and *Mb*^−/−^ mouse heart tissue (n = 3, p < 0.05). (**d**) Box plot of identified lipid classes displaying the fold change in *Mb*^−/−^ heart tissue compard to WT hearts (n = 3). TAG = triacylglycerol, CL = Cardiolipin, PG = Phosphatidylglycerol, PE = phosphatidylethanolamine, DG = diacylglycerol

Next, we characterized the lipid composition using direct infusion MS and MS/MS analysis (Fig. 2a). In terms of the total lipid changes (Fig. 2b) there was a profound increase in specific triglycerides with different FAs containing 16, 18, 20 or 22 carbon atoms in myocardial tissue of the *Mb*^−/−^ mice (Fig. 2c). Particularly oleic acid (C18:1) and palmitic acid (C16:0) tend to be mainly incorporated into triglycerides (Fig. 2c). On the contrary, the phospholipid content comprising the sum of cardiolipin, phosphatidylcholine, phosphatidylinositol, and phosphatidylserine was comparable in *Mb*^−/−^ and WT mouse hearts while phosphatidylethanolamine and phosphatidylglycerol showed a slight decrease in *Mb*^−/−^ mouse hearts (Fig. 2d).

### Loss of myoglobin circumvents channeling of FA to *β*-oxidation

**Figure 3:**
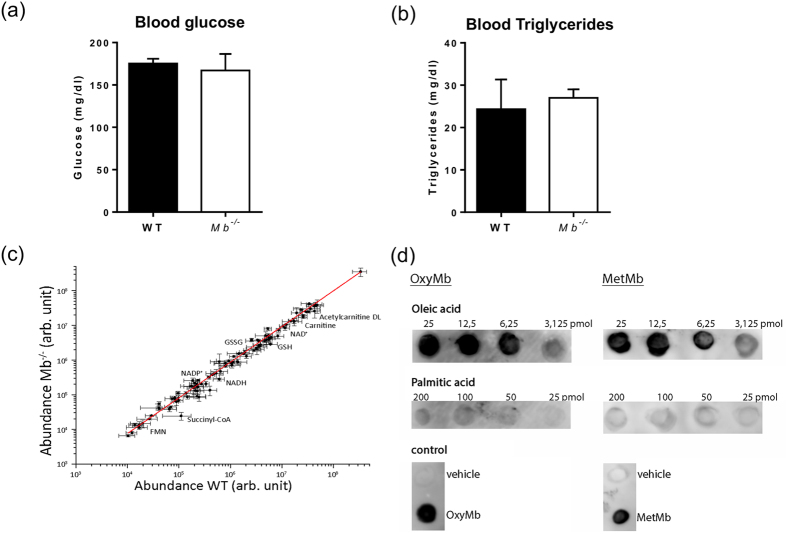
Loss of myoglobin circumvents channeling of FAs to *β*-oxidation. (**a**) Analysis of blood glucose (mg/dl) and (**b**) serum triglycerides (mg/dl) in wild-type (WT) and myoglobin-deficient (*Mb*^−/−^) mouse blood (Values are mean ± SD, n = 5). (**c**) Linear fit of the identified metabolites in mouse heart tissue (*Mb*^−/−^ *vs*. WT) (n = 3). (**d**) Nitrocellulose membrane spotted with oleic and palmitic acid and incubated with oxygenated (oxy) as well as metmyoglobin (MetMb). Bound myoglobin detected with anti-myoglobin antibody.

Recent studies provide evidence that in addition to an increased circulating FA supply an impaired utilization of FAs in the face of continued FA import could also be causative^16^,^17^,^18^,^19^,^26^. If myoglobin contributes to the solubility and the channeling of FA to *β*-oxidation, transgenic mice should exhibit no alterations in blood substrate concentration and no disturbed *β*-oxidation compared to WT mice. Determination of circulating glucose and triglyceride levels revealed comparable levels for these compounds between the 2 groups (glucose: WT mice: 177.5 ± 8.3 mg/dl vs. *Mb*^−/−^ mice 160 ± 20 mg/dl, p = ns; triglycerides: WT mice 29 ± 2.8 *vs. Mb*^−/−^ mice 27 ± 1.6 mg/dl, p = ns; Fig. 3a,b). This is also reflected in comparable blood glucose and serum fatty acid concentrations in 3-month-old *Mb*^−/−^ and WT mice^6^. Furthermore no changes in tissue metabolites could be observed (Fig. 3c). The targeted metabolite analysis of the *Mb*^−/−^ and WT mouse heart tissue performed on a UltiMate 3000 system coupled to a QTRAP6500 mass spectrometer (Fig. 2a) reveals no significant differences, in particular for acetylcarnitine (1.4-fold down-regulation, p = 0.09), acetyl-CoA (1.2-fold down-regulation, p = 0.1) and carnitine (1.6-fold down-regulation, p = 0.06); solely the NADH level shows a 2.3-fold up-regulation (p = 0.03) (see Supplementary Table S1). These findings suggest that *β*-oxidation itself is not impaired by myoglobin ablation. We furthermore investigated the relative degree of interaction of these FAs with myoglobin given that in hearts lacking myoglobin both palmitic acid and oleic acid are incorporated significantly into elevated triglycerides albeit without being desaturated. As examined by a protein-lipid overlay assay, oxygenated as well as metmyoglobin bind profoundly to both FAs with a higher affinity to oleic acid (Fig. 3d).

### No influence of myoglobin ablation on cardiac transcriptome and proteome profile related to FA metabolism

**Figure 4:**
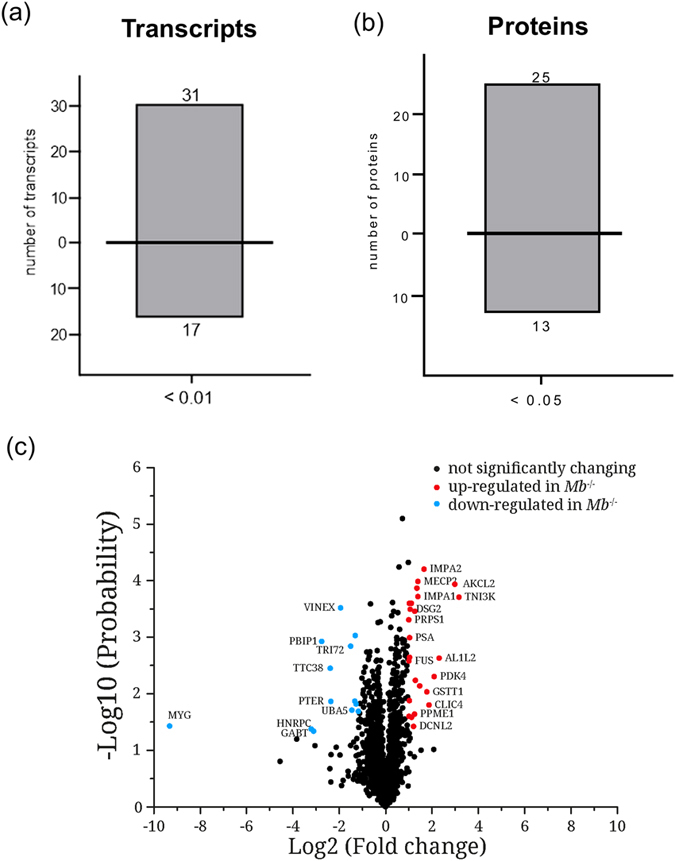
Influence of myoglobin ablation on transcriptome and proteome. (**a**) Differentially expressed cardiac transcripts and (**b**) proteins in wild-type (WT) *vs.* myoglobin deficient (*Mb*^−/−^) mice (Values are mean ± SD, p < 0.01 and p < 0.05, n = 3). (**c**) Volcano plot of 1884 quantified proteins. Red dots classify significantly up- and light blue dots significantly down-regulated proteins in myoglobin-deficient (*Mb*^−/−^) mouse hearts compared to wild-type (WT) mouse hearts. Black dots reflect no significantly changes (n = 3).

Altered regulation of cardiac genes and proteins can be a sensitive predictor of the covert impaired cardiac FA metabolism. The degradation of FAs by *β*-oxidation takes place in the mitochondrial matrix. Long-chain acyl Coenzyme A (CoA) esters converted from FAs by the acyl-CoA synthetase in the cytoplasm are transported *via* the mitochondrial membranes by the carnitine palmitoyltransferase (CPT) system into the matrix. Four enzymes, acyl-CoA dehydrogenase, enoyl-CoA hydratase, L-3-hydroxyacyl-CoA dehydrogenase, and 3-ketoacyl-CoA thiolase orchestrate the *β*-oxidation. The existing various isoforms exhibit each specific affinity for different FA chain-length^12^. The genes encoding key enzymes involved in FA uptake, cytosolic FA binding and esterification, mitochondrial FA uptake and degradation, mitochondrial FA export and glucose metabolism are highly controlled through transcriptional mechanisms^27^,^28^. The most well known regulators are the peroxisome proliferator-activated receptors (PPARs) α and δ, which are abundantly expressed in the myocardium^29^,^30^. Young mouse hearts lacking myoglobin exhibit a 1.4-fold down-regulation of PPARα transcript level^6^. They also show a reduced transcriptional and translational expression of isoforms of the short-chain acyl-CoA dehydrogenase (transcript: 1.4-fold; protein ≈1.5-fold) and short-chain enoyl-CoA hydratase (transcript: 1.5-fold; protein: 1.3-fold and 1.6-fold). The protein expression of L-3-hydroxyacyl-CoA dehydrogenase was also found to be down-regulated by 1.3-fold^6^. On the contrary, these mouse hearts do not accumulate lipids (see Supplementary Fig. S1). We therefore examined the cardiac transcriptome by using the Affymetrix Mouse Transcriptome 1.0 microarray and the proteome by nano-LC/NSI MS/MS in 8-month-old *Mb*^−/−^ mice (Fig. 2a). Deficiency of myoglobin provoked significant changes of 6,246 transcripts with a total of 3,371 up-regulated and 2,875 down-regulated transcripts (uncorrected p-value <0.05). Considering an uncorrected p-value <0.01 and a 2-fold change we revealed significant alterations of 48 transcripts (Fig. 4a). Functional annotation using the Gene Set Enrichment Analysis (GSEA), an advanced computational method utilizing the Molecular Signatures Database (MSigDB, v5.0) which contains more than 8,000 gene sets, suggest an effect of myoglobin deficiency on signaling pathways involved in the fatty acid metabolism. Supplementary Figure S2 depicts affected pathways in mouse hearts lacking myoglobin. Among the pathways with the highest enrichment score was “Fatty acid metabolism” in all three pathway databases analyzed (KEGG, Reactome, Biocarta) as well as “PPAR signaling” in the KEGG and in the Reactome pathway database.

Regarding the exact fold-changes, a significantly altered regulation of the PPARα gene and genes encoding enzymes involved in the FA metabolism, in particular in the degradation by *β*-oxidation occurred in the range of 1.1 to 1.4-fold (See Supplementary Table S2). In general such fold-changes do not reflect any functional impact and could be due to noise. These results were partially confirmed by the proteome analysis. Considering a 2-fold change as a worthwhile cutoff for proteomic studies using nano-LC/NSI MS/MS (Fig. 2a), 25 proteins were significantly up-regulated and 13 proteins down-regulated (Fig. 4b,c and see Supplementary Table S3). Related to the FA metabolism we found the very-long chain enoyl CoA reductase (2.1-fold) and the very-long chain 3-hydroxyacyl-CoA dehydrase (2.4-fold) to be up-regulated. Although myoglobin lacking hearts favor a higher glucose consumption gene expression of pyruvate dehydrogenase kinase (*Pdk*) 4 is 2.5-fold up-regulated and protein expression by 4.3-fold. PDK4 inhibits pyruvate dehydrogenase leading to less oxidation of pyruvate derived from glycolysis^31^,^32^. Glycerol also degraded to pyruvate seems to be dispensable under physiological conditions but becomes important in cardiac stress situations^33^.

### Myoglobin deletion causes depressed cardiac function and atrophy

**Figure 5:**
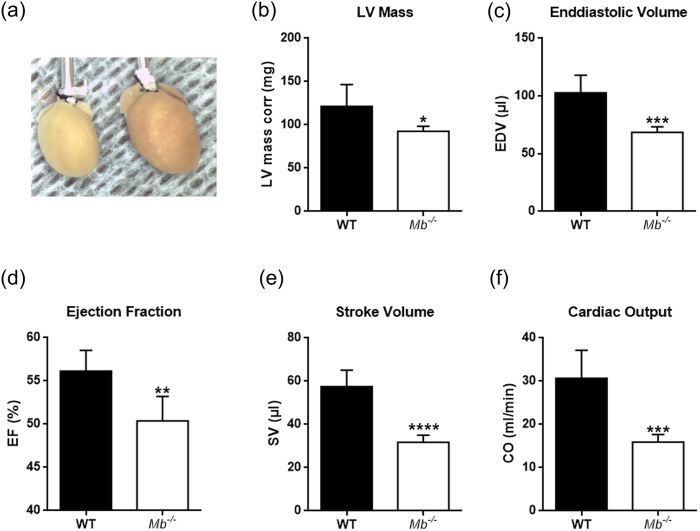
Mouse hearts lacking myoglobin exhibit cardiac dysfunction and atrophy. (**a**) Hearts from a myoglobin deficient (*Mb*^−/−^) mouse (left) and wild-type (WT) mouse (right). (**b**) LV dimension–LV mass corr. (**c–f**) Systolic functional measures–end-diastolic volume (EDV), left ventricular (LV) ejection fraction (EF), stroke volume (SV), cardiac output (CO) using echocardiography in WT and *Mb*^−/−^ mouse hearts. Values are mean ± SD, p < 0.05, *n* = 5.

A potential impact of myoglobin ablation and forced cardiac triglyceride accumulation on chamber size and cardiac function was investigated non-invasively by 2D-directed M-mode echocardiography in WT and *Mb*^−/−^ mice. Mouse hearts with myoglobin deficiency and triglyceride overload displayed altered geometry and cardiac function compared to WT mouse hearts. The influence was evident by a considerably smaller LV mass (−24%) and −32% total heart weight (196 ± 6 mg *vs*. 134 ± 7 mg) with comparable body weights in both groups (~45 g) (Fig. 5a,b and Table 1). However, septal and LV posterior wall thickness at diastole and systole were identical (Table 1). Calculation of the end-diastolic volumes (EDV) revealed a significant decrease by −39% and −22% with respect to LV mass, indicating chamber dilation (Fig. 5c and Table 1). End-systolic volumes (ESV) were decreased by −38% (Table 1); related to LV mass ESV was not different in both groups. Systolic functional measures reveal a reduction of LV ejection fraction (EF) by −10%, stroke volume (SV) by −45% and cardiac output (CO) by −48% in mice lacking myoglobin while heart rate, fractional shortening, E/A, iso-volumetric relaxations time and deceleration time remained comparable (Fig. 5d–f and Table 1).

## Discussion

**Figure 6:**
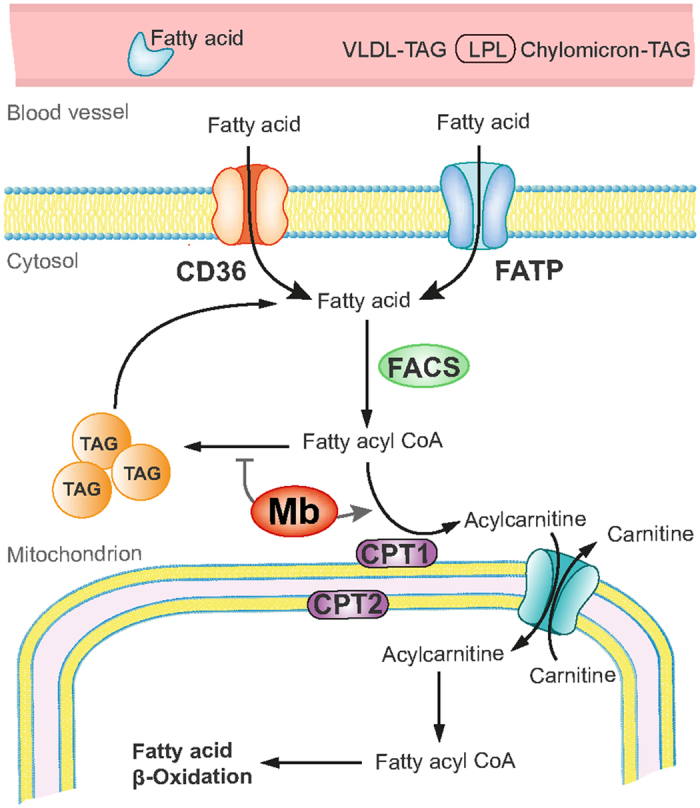
Potential role of myoglobin in cardiac fatty acid metabolism. The heart is supplied with fatty acids (FA) derived from plasma FAs bound to albumin or incorporated in very-low-density lipoproteins (VLDL) triacylglycerols (TAG) or chylomicrons. The uptake occurs *via* diffusion or CD36/FATP transporters. Within the cytosol FAs are converted to FA acyl CoA by fatty acyl coA synthetases (FACS). The majority of FA acyl CoA is then channeled to carnitine palmitoyltransferase (CPT) system mediated by myoglobin (Mb). Converted to acylcarnitine by CPT1 FAs are translocated into the mitochondrial matrix, where they are converted back to fatty acyl CoA by CPT 2 to enter the *β*-oxidation cycle. A minor portion of FA acyl CoA is converted to TAG under the control of Mb.

Myoglobin is critical in O_2_ and NO metabolism regulating a number of important biological processes, including left ventricular function, myocardial short-term hibernation, hypoxic vasodilation and respiratory protection. A recent study using *Mb*^−/−^ mice identified that lack of myoglobin causes an essential shift from FA to glucose oxidation^6^. We here unravel a novel role of cardiac myoglobin in governing FA metabolism to ensure the physiological energy production through *β*-oxidation *via* allocating FAs and preventing intra-myocardial triglycerides accumulation (Fig. 6). Our data reveal that ablation of myoglobin significantly disrupts cardiac function in triglyceride-overloaded 8-month-old hearts and that FA supply as well as the *β*-oxidation itself tend to be unaffected by the loss of myoglobin. Young *Mb*^−/−^ mouse hearts do not exhibit lipid accumulation pointing to a temporally delayed response potentially due to failing compensatory mechanisms.

Under basal physiological conditions, the heart essentially utilizes lipids energy generation^34^. Under ‘stress’ conditions (ischemia, hypertrophy and heart failure), however, glucose is the main resource for the production of ATP. The balance between these two paths is of great relevance to the maintenance of myocardial energy homeostasis. Both, a pronounced inhibition of FA oxidation and a maximum increased uptake and utilization of lipids can lead to cardiac dysfunction. The patho-mechanisms through which increased lipids act lipotoxic are incompletely understood^34^.

Fatty acids as the main energy source of the heart derive from triglycerides in the blood, which, in turn, result from both dietary lipid intake as well as from hepatic *de novo* synthesis. Thereby, the respective blood FA levels represent the primary determinant of the rate of myocardial lipid uptake. In obesity and diabetes, circulating FA levels are chronically elevated and entail high rates of uptake^12^. Our *Mb*^−/−^ mouse model does not exhibit any discernable alterations in blood triglyceride levels. Likewise, the ablation of myoglobin only provokes a 1.15-fold up-regulation of lipoprotein lipase (LPL), which is essential for hydrolysis of triglycerides - a critical step for their uptake. High LPL expression in the heart also accounts for a comparatively high amount FA uptake^35^,^36^,^37^. This holds particularly true for the adult heart, but not for the neonatal period, which is characterized by an increased use of cardiomyocytes glucose for energy^37^. These data suggest that the accumulation process is determined by intracellular mechanisms such as reduced *ß*-oxidation capacity by down-regulation of genes encoding involved proteins. This is apparent in young *Mb*^−/−^ mouse hearts exhibiting a down-regulation of PPARα (1.4-fold), and of acyl-CoA dehydrogenase (1.4-fold) and enoyl-CoA hydratase (1.5-fold) isoform transcripts. The latter are down-regulated on the translational level by ≈ 50% and 33%, respectively^6^. Gene and protein expression as well as metabolome analyses in the 8-month-old *Mb*^−/−^ mouse do not reveal appreciable changes relating to FA metabolism suggesting a diminished but not disturbed *ß*-oxidation. This points to a cytosolic origination of lipid deposition. Myoglobin is localized in the cytoplasm and abundant with 200–300 nmol/g wet weight. Mathematical models as well as *in vitro* studies have previously suggested that intracellular FA carriers would be indispensable for trans-sarcoplasmic transport^38^. In this respect, FABP is widely acknowledged as an important intracellular FA carrier. Rat cardiac FABP accounts for approximately 3% of the cytoplasmic proteins (50 nmol / g wet weight)^39^, which is 4–6-fold lower compared to myoglobin. At least *in vitro*, H-FABP can bind to various fatty acid types (e.g., palmitate, oleate, stearate, linoleate, and arachidonate)^40^. Here we show that oxygenated and metmyoglobin bind to palmitic and oleic acid with a higher affinity to oleic acid. Both FAs were found to be mostly incorporated into the accumulated triglycerides. Studies regarding the physiological relevance of H-FABP in FA handling in isolated cardiomyocytes of mice carrying a targeted disruption of the gene coding for H-FABP unmasked in lieu thereof a role in FA uptake. This was demonstrated by a markedly lowered uptake rate of palmitate (−250%) and a significantly depressed palmitate oxidation despite the fact that the capacity for FA oxidation was similar in H-FABP knock-out and WT mice^41^,^42^. Intriguingly, *Mb*^−/−^ mice exhibit a diminished FA oxidation and an augmented triglyceride accumulation. We speculate that myoglobin enhances substantially the solubility of fatty acids in the sarcoplasm and mediates channeling to *ß*-oxidation.

Lipid-associated cardiotoxicity is based on a dysregulation of apoptosis, inflammation, mitochondrial dysfunction and intracellular signaling pathways^34^. The exact interaction and relative contribution of each individual component is not known. Cardiac dysfunction is closely associated with induction of cellular apoptosis^2^,^43^ and lipids seem to play a crucial role in particular in the phase of apoptosis induction. Elevated palmitate levels increase, e.g., the formation of ROS, induce a cytochrome c release into the cytoplasma, lead to mitochondrial cardiolipin loss and finally cause an alteration of mitochondrial structures. The intracellular signaling pathways that can be affected by lipids include the activation of protein kinase C (PKC), mitogen activated protein kinases (MAPK; among other Erk, JNK, p38) and, the PPAR family^44^,^45^,^46^. Particularly the PPAR family is now regarded as the master regulator of lipid metabolism. PPAR family members contribute, amongst others, to an adequate activation of enzymes of fatty acid oxidation. On the other hand, it could be shown that in the failing heart PPAR activity is reduced and that ROS may trigger a down-regulation of PPAR. Whether a reduction of ROS leads to an optimization of the PPAR activity is not known.

The present studied *Mb*^−/−^ mice show that their hearts are affected by an overload of lipids as compared to control hearts. The widely accepted textbook status of myoglobin has recently been challenged by studies that show a distinct involvement of myoglobin in the regulation of NO and ROS signaling under physiological and pathophysiological conditions^2^,^5^,^8^,^47^,^48^,^49^. Myoglobin-derived NO signaling closely regulates mitochondrial energetics and thus adjusts myocardial function to a reduced O_2_ supply. This is in part accomplished by a reduction in ROS derived from mitochondrial complexes or from an increased ROS decomposition. Under absence of myoglobin, lipids are accumulated in the myocardium accompanied with a dysregulation of lipid signaling molecules. While these signaling components tightly interact with and are regulated by ROS, it is tempting to speculate whether myoglobin impacts lipid signaling by regulation of ROS levels.

### Limitations

We assessed cardiac function in these mice using high-resolution ultrasound, which showed a markedly impaired heart function. This refers mainly to a reduced stroke volume, ejection fraction and the resulting cardiac output. However, *Mb*^−/−^ mice and WT controls do not differ in body weight and heart rates. In further studies, it will be of high importance to relate this to an impaired cardiopulmonary exercise capacity, which is a limitation of the current examinations. Also, this present investigation does not include a functional assessment of the complex lipid metabolism, which must a subject of future trials albeit on the cellular (cardiomyocyte) and mitochondrial levels.

Diabetic cardiomyopathy is a pronounced disturbance of lipid utilization in humans. Experimental studies suggest that this distinct cardiomyopathy is characterized by lipid accumulation, disturbance of cardiomyocyte function and, subsequently, sudden death, without concurrent coronary heart disease. A therapeutic option for the prevention of this cardiomyopathy is therefore essential. We here show that lack of cardiac myoglobin is associated with an accumulation of lipids in the heart. This, in turn, goes along with a substantially disturbed cardiac function. It is presently not known, whether patients with cardiac lipid metabolism impairment also suffer from a reduced myoglobin expression. Future studies must investigate, whether the present mechanism may provide a clinically relevant option to treat lipotoxicity in the heart.

## Methods

### Mice and ethics statement

*Mb*^−/−^ mice were generated by ablation of the essential exon-2 *via* homologous recombination in embryonic stem cells as described^50^ and were bred at the central animal facility of the Heinrich Heine University (Düsseldorf, Germany). NMRI mice were obtained from Janvier (Saint Berthevin, France) and kept one week in the local animal house for acclimatization. Mice utilized in the present studies were at 32±3 weeks with a body weight of 44 ± 2 g and 12 ± 3 weeks with a body weight of 28 ± 2 g for comparison^6^. Mice were kept on a standard rodent chow containing fatty acids (C14:0–0.01%; C16:0–0.68%; C16:1–0.04%; C18:0–0.22%; C18:1–1.44%; C18:2–3.21%; C18:3–0.37%; C20:0–0.03%; C20:1–0.01%) prior to experimental use. All experiments were approved by the responsible committee (LANUV, Recklinghausen, Germany) according to the ‘European Convention for the Protection of Vertebrate Animals used for Experimental and other Scientific Purposes’ (Directive 2010/63/EU) and animal care was in accordance with institutional guidelines.

### Lipid imaging

To visualize the accumulation of lipids in the mouse hearts, 8-μm frozen sections were fixed with 10% paraformaldehyde and then stained with Oil-Red-O solution (Sigma, Darmstadt, Germany) (n = 3). Tissue sections were counterstained with Mayers hematoxylin solution (Sigma). Photomicrographs of stained sections were taken on an Olympus BX51 microscope^51^.

### Cardiac ^1^H-MRS

Data were recorded on a Bruker Avance^III^ 9.4 Tesla Wide Bore (89 mm) nuclear magnetic resonance (MR) spectrometer essentially as described^6^,^52^,^53^. Experiments were carried out using a Bruker microimaging unit (Micro 2.5) equipped with actively shielded gradient sets (capable of 1.5 T/m maximum gradient strength and 150 μs rise time at 100% gradient switching), a 25-mm birdcage resonator, and Paravision 5.1 as operating software. The mice were anesthetized with 1.5% isoflurane and were kept at 37 °C. The front paws and the left hind paw were attached to ECG electrodes (Klear-Trace; CAS Medical Systems, Branford). Respiration was monitored by means of a pneumatic pillow positioned at the animal’s back. Vital functions were acquired by a M1025 system (SA Instruments, Stony Brook, NY, USA) and used to synchronize data acquisition with cardiac and respiratory motion. For functional analysis, high-resolution images of mouse hearts were acquired in short axis orientation using a ECG- and respiratory-gated segmented cine fast gradient echo cine sequence with steady state precession (FISP). A flip angle of 15°, echo time (TE) of 1.23 ms, and a repetition time (TR) of about 6–7 ms (depending on the heart rate) were used to acquire 16 frames per heart cycle. The pixel size after zero filling was 117 × 117 μm^2^ (field of view, 30 × 30 mm^2^; acquisition time per slice for one cine sequence, ~1 min). Eight to ten contiguous slices were acquired to cover the entire heart.

For quantification of cardiac lipids, a 1 × 2 × 3 mm^3^ voxel was placed in the septum as shown in Fig. 1A. Fieldmap-based shimming (MAPSHIM) was carried out to optimize the field homogeneity in the region of interest followed by manual shimming. ^1^H MR spectra were acquired using ECG- and respiratory-gated single-voxel point resolved spectroscopy (PRESS) with a chemical shift selective (CHESS) water suppression module and outer volume suppression (OVS). The following parameters were used: TR, 1000 ms; TE, 9.1 ms; averages, 1024; data points in the spectral domain, 256; spectral width, 5000 Hz; acquisition time, 51.2 ms; an exponential filter of 10 Hz was applied and chemical shifts were referenced to the prominent methylene (-CH_2_-) peak in the water-suppressed spectra at 1.3 ppm. The exact repetition time of the sequence was determined by the heart rate with physiologically derived steady state maintenance during respiration. Total preparation time including CHESS (73.23 ms) and OVS (18.63 ms) was 91.86 ms. Localized acquisition was timed at ~40% of the cardiac cycle in systole to maximize tissue thickness and homogeneity, typically requiring a trigger delay of 60 to 80 ms after ECG R-wave upslope detection. Water-suppressed and unsuppressed cardiac spectra from same voxel positioned in the septum were acquired in using the PRESS sequence with and without CHESS suppression. To quantify the myocardial metabolite content, the integral of the lipid signal was divided by the corresponding integral of the water signal from unsuppressed spectra, thus reporting lipid content as a percentage of the water signal.

### SIMPLEX (SImultaneous Metabolite, Protein, Lipid EXtraction procedure) protocol

After grinding in liquid nitrogen, heart tissue powder was weighed in pre-tared cooled vials and the SIMPLEX workflow was employed as previously described^54^. Briefly, cold MeOH was added to the grinded cardiac tissue, vortexed for 20 s, and incubated in liquid nitrogen for 1 min. Further, the samples were allowed to thaw at RT and were sonicated for 10 min at 4 °C. This procedure was repeated three times. Next, cold MTBE was added and the mixture was incubated for 1 h at 4 °C in a thermomixer. Phase separation was induced by adding 188 μL water containing 0.1% ammonium acetate. The extract was centrifuged at 10,000 *g* for 5 min and the upper phase was collected, dried under nitrogen flow and dissolved in 200 μL 2-Prop/MeOH/CHCl_3_ (4:2:1, v/v/v) containing 7.5 mM ammonium acetate for further analysis. For protein precipitation, MeOH was added to the remaining lower phase in a final ratio of 4:1, v/v MeOH/H_2_O, and the samples were incubated for 2 h at −80 °C, followed by 30 min centrifugation (13,000 *g)* at 4 °C. The resulting supernatant was removed and the remaining pellet dissolved in 1% SDS, 150 mM NaCl, 50 mM Tris (pH 7.8) for further label free proteomics experiments. The dried metabolite extracts were resuspended in 200 μL ACN/H_2_O (9:1, v/v), centrifuged for 10 min (13,000 *g*) at 4 °C to remove any insoluble debris and then stored at −80 °C prior to LC/MS analysis.

### Proteomics sample preparation

Bicinchoninic acid assay (Pierce, Thermo-Fisher, Bremen, Germany) was performed to determine protein concentrations. The disulfide bonds were reduced by 30 min incubation at 56 °C with 10 mM DTT, and free sulfhydryl groups were alkylated using 30 mM IAA for 30 min at RT in the dark. Samples containing 100 μg protein were processed using FASP (Filter Aided Sample Preparation)^55^,^56^, with a 30 kDa molecular weight cut-off spin filter. The digestion was performed in 50 mM TEAB, 0.2 M GuHCl, 2 mM CaCl_2_, using a trypsin to protein ratio of 1:25 (w/w) for 12 h at 37 °C^57^. Peptides were eluted from the filter by centrifugation, with 50 mM TEAB and acidified to a final concentration of 1% TFA. Digestion efficiency was controlled by monolithic RP separation and the samples were stored at −80 °C^58^.

### Lipid analysis, direct infusion MS and MS/MS analysis

The lipid extracts were diluted in 2-Prop/MeOH/CHCl_3_ (4:2:1, v/v/v) with 7.5 mM ammonium acetate in a 96 well plate (Eppendorf, Hamburg, Germany) and then infused *via* robotic nanoflow ion source TriVersa NanoMate (Advion BioSciences, Ithaca NY, USA) into a Q Exactive Plus instrument (Thermo Fisher Scientific, Bremen, Germany) using chips with spraying nozzles of 4.1 μm. The ion source was controlled by Chipsoft 8.3.1 software (Advion Biosciences). Ionization voltage was set to +1.25 kV in positive and −1.25 kV in negative mode and the backpressure was set at 0.95 psi in both modes. The s-lens level was 60% and the temperature of the ion transfer capillary was adjusted to 250 °C. Polarity switch of the TriVersa NanoMate was triggered by the mass spectrometer via contact closure signal as described previously^59^.

For data-dependent experiments (DDA), full MS spectra were acquired under the target mass resolution R_m/z 200_ of 140 000 (full width at half maximum at m/z 200). Precursors were selected within the m/z window of 1.2 Da and the fragment ions were detected in the Orbitrap mass analyzer using a target mass resolution R_m/z 200_ of 35 000 FWHM. All spectra were imported by LipidXplorer software into a MasterScan database under the following settings: mass tolerance 5 ppm; range of m/z 320–1200; min occupation of 1; intensity threshold 1 × 10^4^ and lipid identification was carried on as described by Herzog *et al*.^60^,^61^.

### Metabolite Analysis, LC-MS/MS

The metabolite analyses were performed on an UltiMate 3000 system coupled to a QTRAP 6500 mass spectrometer (AB SCIEX). For separation, hydrophilic interaction liquid chromatography (HILIC) was carried out on a Zic^®^ -HILIC column (150 × 1 mm, 3.5-μm particle size, 100 Å pore size) from Merck (Darmstadt, Germany). The mobile phases were 90% acetonitrile (A) and 20 mM ammonium acetate, pH = 7.5 in H_2_O (B). The gradient eluted isocratically with 90% ACN for 2.5 min followed by an increase to 60% over 14 min and held at 60% for 2 min. Subsequent reconstitution of the starting conditions and re-equilibration with 100% A for 10 min resulted in a total analysis time of 35 min. 2 μL of sample were injected onto the column and the LC separation was carried out at 25 °C under a flow rate of 100 μL min-1. ESI Turbo V source parameters were set as follows: curtain gas, 30 arb. unit; temperature, 350 °C; ion source gas 1, 40 arb. unit; ion source gas 2, 65 arb. unit; collision gas, medium; ion spray voltage, 5500 V/−4500 V (positive mode/ negative mode). For the selected reaction monitoring (SRM) mode Q1 and Q3 were set to unit resolution and the transitions for all compounds were adapted^62^,^63^. For data acquisition and analysis Analyst (1.6.2) and MultiQuant 3.0 were used respectively.

### Lable free proteomics

Samples were separated on an Ultimate 3000 Rapid Separation Liquid Chromatography (RSLC) system (Dionex, Thermo Fisher Scientific) coupled to a Q Exactive Plus, using data dependent MS/MS acquisition. Peptides were preconcentrated on a 100 μm ID trapping column (Acclaim C18 PepMap100, 100 μm x 2 cm, Thermo Scientific) followed by separation on a 75 μm ID RP main column (Acclaim C18 PepMap100, 75 μm x 50 cm, Thermo Scientific) using a binary gradient (solvent A: 0.1% FA and solvent B: 0.1% FA in 84% ACN) at a flow rate of 250 nL/min. The gradient increased linearly from 5% to 45% B over 200 min.

For the Q Exactive analysis, full MS scans were acquired at a resolution of 70 000 FWHM, followed by MS/MS of the 15 most abundant ions at 17 500 FWHM. Target value and maximum injection time were set to 3 × 10^6^ ions and 120 ms for the full scan and 5 × 10^4^ ions and 250 ms for MS/MS scans. Only precursors with charge states between +2 and +5 were selected for fragmentation.

### Blood parameters

Evaluation of triglycerides in blood serum was performed by the Central Laboratory of the University Hospital Essen using clinical routine protocols. Glucose levels were determined using the Accu-Chek Aviva blood glucose meter system (Roche Diabetes Care, Mannheim, Germany). Mice were starved for 3 h. (n = 5)

### Protein-lipid overlay assay

A protein–lipid overlay assay was performed using recombinant horse heart myoglobin (Sigma) (n = 3). Lipid solution (2 μl) containing 3.12–25 pmol of oleic acid and 25–200 pmol of palmatic acid dissolved in chloroform was spotted onto iBind Stack- membranes (ThermoFisher, Schwerte, Germany) and dried after infiltration at room temperature (RT) for 5 min. The membrane was washed in Tris-buffered saline containing 0.05% Tween-20 (TBS-T) for 10 min at RT. Membranes were incubated for 10 min at RT on a shaker with oxygenated and metmyoglobin solution (200 μM). Oxygenated and metmyoglobin solutions were prepared according to an established protocol^46^,^47^. After washing, membranes were blocked with 5% bovine serum albumin (BSA) in TBS-T for 2 h and then incubated overnight at 4 °C with an anti-myoglobin antibody (Santa Cruz, Heidelberg, Germany; catalog-number sc-25607; 1:1,000 dilution in TBS-T containing 0.1% BSA). Membranes, washed two times in TBS containing 0.1% Tween-20, were incubated for 1 h with horseradish peroxidase-conjugated goat anti-rabbit antibody (ThermoFisher, catalog-number 32260; 1:2,000 dilution) at RT. The membranes were washed two times at RT in TBS containing 0.1% Tween-20 at RT, and signals were detected by enhanced chemiluminescence (ThermoFisher).

### Echocardiography

Echocardiography was performed using a Vevo 2100 High-Resolution Imaging System (VisualSonics, Toronto, Canada) equipped with a 40 MHz ultrasound probe. Data analysis was assessed off-line with dedicated Vevo Analytic Software. Briefly, mice were anesthetized with 3% isoflurane, hair was removed from the thorax and animals were put under light anesthesia (1.5% isoflurane) at 37 °C. After animals were positioned supine with limbs taped to electrocardiogram (ECG) electrodes incorporated into a temperature-controlled printed circuit board, two-dimensional, M-mode, pulse wave and tissue Doppler transthoracic echocardiography was performed to assess chamber dimensions, systolic and diastolic functions in means of LV mass, IVS and LVPW thickness, EF, SV, CO, EDV, ESV, HR, FS, E/A, IVRT, and DT. Pulse Doppler images were collected with the apical four-chamber view with the sample volume moved slightly toward the left ventricular outflow tract to intersect with both the mitral inflow and the left ventricular outflow in the same recording. Early (E) and late (A) diastolic mitral inflow pattern were determined, as well as the left ventricular systolic and diastolic time intervals. Tissue Doppler imaging was performed in the apical four-chamber view with the sample volume set to measure at the septal side of the mitral annulus.

### Gene expression analyses

The preparation of total RNA from hearts was performed hearts using the RNeasy Midi Kit (Qiagen, Hilden, Germany). The extraction procedure was conducted in accordance with the manufacturer’s manual. RNA integrity was checked with RNA Nano LabChips on an Agilent 2100 Bioanalyzer (Agilent Technologies, Palo Alto, CA). All samples in this study showed high quality RNA Integrity Numbers (RIN; mean 8.9). RNA was further analyzed by photometric Nanodrop measurement and quantified by fluorometric Qubit RNA assays (Life Technologies).

Synthesis of biotin labeled cDNA was conducted on three replicates of both experimental groups according to the manufacturers´ protocol (WT Plus Reagent Kit; Affymetrix, Inc). Briefly, 200 ng of total RNA were converted to cDNA. After amplification by *in vitro* transcription and another cDNS synthesis cycle, cDNA was fragmented and biotin labeled by terminal transferase. Finally, cDNA was hybridized to Affymetrix Mouse Transcriptome 1.0 arrays, washed and stained and scanned on the GC Scanner 3000 with G7 update as described in the standard Affymetrix GeneChip protocol (Version 2). Signal summarization using the RMA algorithm as well as statistical analysis were performed using the Affymetrix TAC software. The significance threshold was set to p < 0.05 and 0.01.

### Label free data analysis

Label free quantification was performed using the Progenesis LC-MS software version 4.1 from Nonlinear Dynamics (Newcastle upon Tyne, U.K.). MS raw data were aligned in automatic mode. Exported peak lists were searched using searchGUI 3.0.3^64^, Mascot 2.4 (Matrix Science) and X!Tandem Vengeance (2015.12.15.2), using a concatenated target/decoy version of the mouse Uniprot database (downloaded on 22nd of July 2015, containing 33432 target sequences). Data analysis was done using PeptideShaker 0.28.0 (http://compomics.github.io/projects/peptide-shaker.html), to maximize number of identified peptides and proteins. The following search parameters were used: trypsin as protease with a maximum of two missed cleavages; carbamidomethylation of Cys as fixed and oxidation of Met as variable modification. MS and MS/MS tolerances were set to 10 ppm and 0.02 Da. In PeptideShaker^65^ search results were combined and filtered at a false discovery rate of 1% on the protein level prior to export and re-import into Progenesis. Peptide sequences containing oxidized Met and pyro-Glu (derived from X!Tandem 2nd pass search) were omitted from further data analysis. Only proteins with at least two unique peptides were considered for quantification. For each protein, the average of the normalized abundances (obtained from three replicates processed with Progenesis) was calculated for the comparison of *Mb*^−/−^ and WT mouse hearts. As regulated were considered only those proteins which were (i) commonly quantified in all six samples with (ii) at least two unique peptides, (iii) an p-value of <0.05 and (iv) an average ratio <0.5 or >2 (corresponding to 2-fold regulation).

### Bioinformatics analyses

Functionally relevant pathways were identified using GSEA software^66^,^67^. In the In the GSEA analysis all genesets provided by MSigDB (v5.0) were analyzed using default settings.

### Statistical analyses

Results are expressed as means ± SD. Unpaired Student’s *t* test was used as appropriate. p < 0.05 was considered statistically significant.

## Additional Information

**How to cite this article:** Hendgen-Cotta, U. B. *et al*. A novel physiological role for cardiac myoglobin in lipid metabolism. *Sci. Rep.* **7**, 43219; doi: 10.1038/srep43219 (2017).

**Publisher's note:** Springer Nature remains neutral with regard to jurisdictional claims in published maps and institutional affiliations.

## Supplementary Material

Supplementary Information

## Figures and Tables

**Table 1:** LV mass systolic and diastolic parameter in *Mb* ^−/−^ *vs.* WT mice.

Parameter	Units	Mice	Value	SD	p-Value
LV mass	mg	WT	121.3	25.2	0.0328*
		*Mb*^−/−^	92.5	5.9	
EF	%	WT	56.1	2.4	0.0035**
		*Mb*^−/−^	50.4	2.8	
SV	μl	WT	57.6	7.6	<0.0001****
		*Mb*^−/−^	31.7	3.3	
CO	ml/min	WT	30.7	6.5	0.0006***
		*Mb*^−/−^	15.9	1.8	
EDV	μl	WT	102.9	15.1	0.0007***
		*Mb*^−/−^	68.6	4.7	
ESV	μl	WT	45.3	8.2	0.0014**
		*Mb*^−/−^	28.2	3.5	
Fractional shortening	%	WT	8.8	5.2	ns
		*Mb*^−/−^	11.4	2.4	
Heart Rate	bmp	WT	510	31	ns
		*Mb*^−/−^	488	11	
Intraventricular septum, diastolic (d)	mm	WT	0.8	0.2	ns
		*Mb*^−/−^	0.9	0.1	
Intraventricular septum, systolic (s)	mm	WT	1.0	0.2	ns
		*Mb*^−/−^	1.0	0.1	
Left Ventricular Posterior Wall, d	mm	WT	0.9	0.2	ns
		*Mb*^−/−^	0.8	0.2	
Left Ventricular Posterior Wall, s	mm	WT	1.2	0.1	ns
		*Mb*^−/−^	1.1	0.0	
E/A		WT	2.2	0.7	ns
		*Mb*^−/−^	2.3	0.9	
Isovolumetric Relaxation Time	ms	WT	14.1	3.9	ns
		*Mb*^−/−^	18.1	1.3	
Deceleration Time	ms	WT	12.3	0.5	ns
		*Mb*^−/−^	12.2	1.4	
