# Supplementary material for: A novel physiological role for cardiac myoglobin in lipid metabolism
Source: Sci Rep. 2017 Feb 23;7:43219. doi: 10.1038/srep43219 (PMC5322402; doi:10.1038/srep43219)
Supplement: Supplementary Information [file srep43219-s1.pdf]

**Supplementary Information**

# A novel physiological role for cardiac myoglobin in lipid metabolism

Ulrike B. Hendgen-Cotta[1], Sonja Esfeld[1], Cristina Coman[2], Robert Ahrends[2], Ludger Klein-Hitpass[3], Ulrich Flögel[4], Tienush Rassaf[1], and Matthias Totzeck[1,*]

[1]University Hospital Essen, Medical Faculty, West German Heart and Vascular Center, Department of Cardiology and Department of Angiology, Hufelandstr. 55, 45147 Essen, Germany

[2]Leibniz-Institut für Analytische Wissenschaften – ISAS e.V. Otto-Hahn-Str. 6b, 44227 Dortmund, Germany

[3]University Hospital Essen, Institute of Cell Biology, Medical Faculty, Virchowstr. 173, 45122 Essen, Germany

[4]University Hospital Düsseldorf, Department of Molecular Cardiology, Universitätsstr. 1, 40225 Düsseldorf, Germany

*Corresponding author
Dr. med. Matthias Totzeck
Hufelandstr. 55, 45147 Essen
Tel. +49 201 723-84805
Matthias.Totzeck@uk-essen.de

**Supplementary Table S1: Metabolites in myoglobin deficient compared to WT mouse hearts**

| Metabolites | Log2 (Fold change) | Log10 (p value) |
|---|---|---|
| Acetylcarnitine DL | -0.5 | 1.0 |
| Acetylcholine | -0.2 | 0.6 |
| Acetyl-CoA | -0.2 | 1.0 |
| Adenine | 0.0 | 0.0 |
| Adenosine phosphosulfate | -0.6 | 1.2 |
| ADP | -0.5 | 1.0 |
| ADP_Rib | -0.7 | 0.5 |
| Alanine | -0.3 | 0.7 |
| AMP | -0.7 | 0.6 |
| Arginine | -0.8 | 0.9 |
| Ascorbic acid | 2.5 | 1.9 |
| Asparagine | -0.3 | 0.6 |
| Aspartic acid | 0.0 | 0.0 |
| ATP | 0.5 | 0.2 |
| Benzoic acid | -0.4 | 0.3 |
| Carnitine | -0.7 | 1.3 |
| Choline | 0.2 | 1.4 |
| Citric acid | 0.5 | 1.2 |
| Citrulline | 0.1 | 0.2 |
| CMP | -0.8 | 1.1 |
| CMPneg | -0.7 | 1.0 |
| Coenzyme A | -1.4 | 0.9 |
| Creatine | -0.4 | 0.9 |
| CTP | 0.4 | 0.5 |
| cyclic AMP | -0.3 | 0.7 |
| Cytosine | 0.0 | 0.0 |
| dAMP | -0.4 | 0.4 |
| dCMP | -0.7 | 0.7 |
| Deoxyadenosine | 0.4 | 0.5 |
| Deoxyguanosine | -0.1 | 0.0 |
| dGDP | -0.5 | 1.1 |
| dGMP | -0.5 | 0.4 |
| DL_Pipecolic acid | -0.6 | 1.1 |
| Erythrose 4_phosphate | -1.7 | 1.3 |
| FAD | -0.2 | 0.8 |
| FMN | -0.7 | 0.5 |
| Fumaric acid | -0.3 | 0.2 |
| Galactose | -0.1 | 0.1 |
| GDP | 0.0 | 0.0 |
| Glucosamine 6_phosphate | -0.8 | 1.2 |
| Glutamic acid | -0.9 | 1.0 |
| Glutamine | -0.6 | 1.2 |
| Glycerol 3_phosphate | -1.1 | 1.0 |
| Glycerophosphocholine | 0.2 | 0.3 |

| | | |
|---|---|---|
| Glycine | -0.1 | 0.4 |
| GMP | -0.7 | 0.5 |
| GSH | -0.5 | 1.0 |
| GSSG | 0.5 | 1.7 |
| Guanine | -0.3 | 0.3 |
| Guanosine | -0.5 | 0.5 |
| Hexose monophosphate | -1.6 | 1.3 |
| IDP | -0.5 | 1.0 |
| IMP | -0.5 | 0.4 |
| indole | -0.1 | 0.0 |
| Inosine | -0.8 | 0.8 |
| Iso_Leucine | -0.3 | 0.4 |
| Lactic acid | -0.8 | 0.5 |
| Lysine | -0.6 | 1.3 |
| Malic acid | -0.3 | 0.2 |
| Malonyl-CoA | -0.4 | 0.8 |
| Methionine | -0.3 | 0.4 |
| methylnicotinamide | -0.5 | 0.6 |
| Myoinositol | -0.1 | 0.0 |
| N_Acetyl_glucosamine 1_phosphate | -0.1 | 0.2 |
| N_acetylserine | 0.1 | 0.2 |
| NAD+ | -0.5 | 0.9 |
| NADH | -1.2 | 1.5 |
| NADP+ | -0.4 | 1.2 |
| Niacinamide | 0.2 | 0.1 |
| Orotidylic | -0.7 | 1.1 |
| Oxoglutaric acid | -0.6 | 1.1 |
| Pantothenic acid | -0.3 | 1.0 |
| Phenylalanine | -0.2 | 0.2 |
| Phosphorylcholine | -0.7 | 0.6 |
| Proline | 0.1 | 0.1 |
| Propionyl-CoA | -0.9 | 0.8 |
| Riboflavin | -0.5 | 0.6 |
| Ribose 5_phosphate | -0.4 | 0.4 |
| S_methyl_5_thioadenosine | -0.6 | 0.7 |
| S-Adenosylhomocysteine | -0.1 | 0.2 |
| S-Adenosylmethionine | -0.5 | 0.6 |
| Sarcosine | -0.3 | 0.6 |
| Sedoheptulose monophosphate | -0.3 | 0.5 |
| Serine | 0.0 | 0.0 |
| Shikimic acid | -0.5 | 0.2 |
| Succinic acid | -0.4 | 0.3 |
| Succinyl-CoA | -2.3 | 0.9 |
| Taurine | -0.5 | 0.9 |
| Thiamine | 0.3 | 0.8 |
| ThiamineP | -0.5 | 0.7 |
| threonine | 0.0 | 0.1 |
| Tryptophan | -0.1 | 0.1 |
| Tyramine | -0.5 | 0.4 |

| | | |
|---|---|---|
| Tyrosine | -0.4 | 0.4 |
| UDP | -0.8 | 2.3 |
| UDP D glucose | -0.7 | 2.1 |
| UDP D glucuronate | -0.3 | 1.3 |
| UDP_n_acetyl_D_glucosamine | -0.5 | 0.4 |
| UMP | -0.9 | 0.8 |
| Uracil | 0.0 | 0.0 |
| Uridine diphosphate glucuronic | -0.3 | 1.4 |
| Valine | -0.4 | 0.7 |
| Xanthine | -0.4 | 0.3 |

**Supplementary Table S2: Genes involved in the fatty acid metabolism in myoglobin deficient *vs.* wild-type mouse hearts**

| Gene | Protein | Fold | P-Value |
|---|---|---|---|
| Acsl1 | acyl-CoA synthetase long-chain family member 1 | 1.2 | 0.027 |
| Cpt1b | carnitine palmitoyltransferase 1b, muscle | 1.1 | 0.042 |
| Cpt2 | carnitine palmitoyltransferase 2 | 1.2 | 0.009 |
| Acadm | acyl-CoA dehydrogenase, medium chain | 1.2 | 0.007 |
| Acadl | acyl-CoA dehydrogenase, long chain | 1.1 | 0.009 |
| Acadvl | acyl-CoA dehydrogenase, very long chain | 1.2 | 0.006 |
| Acads | acyl-CoA dehydrogenase, short chain | 1.1 | 0.047 |
| Ehhadh | enoyl-CoA hydratase/3-OH-acyl CoA dehydrogenase | 1.4 | 0.007 |
| Hadh | 3-OH-acyl-CoA dehydrogenase | 1.1 | 0.018 |
| Hadha | 3-OH-acyl-CoA dehydrogenase/3-ketoacyl-CoA thiolase/enoyl-CoA hydratase, alpha subunit | 1.2 | 0.002 |
| Hadhb | 3-OH-acyl-CoA dehydrogenase/3-ketoacyl-CoA thiolase/ enoyl-CoA hydratase, beta subunit | 1.1 | 0.021 |
| Acaa1a | acetyl-CoA acyltransferase 1A (3-ketoacyl-CoA thiolase) | -1.1 | 0.004 |
| Acaa1b | acetyl-CoA acyltransferase 1B (3-ketoacyl-CoA thiolase) | -1.1 | 0.020 |
| Acaa2 | acetyl-CoA acyltransferase 2 (3-ketoacyl-CoA thiolase, mitochondrial) | 1.3 | 0.002 |
| Decr1 | 2,4-dienoyl CoA reductase 1, mitochondrial | 1.3 | 0.01 |
| Ppara | peroxisome proliferator activated receptor alpha | 1.1 | 0.02 |
| Ppard | peroxisome proliferator activator receptor delta | 1.1 | 0.02 |
| Rxrg | retinoid X receptor gamma | 1.1 | 0.04 |
| Angptl4 | angiopoietin-like 4 | 2.7 | 0.001 |

| | | | |
|---|---|---|---|
| Lpl | lipoprotein lipase | 1.2 | 0.045 |
| Slc27a1 | solute carrier family 27 (fatty acid transporter), member 1 | 1.3 | 0.02 |
| Fabp7 | fatty acid binding protein 7 | 1.3 | 0.01 |
| Scd2 | stearoyl-CoA desaturase 2 | -1.5 | 0.02 |
| Me1 | malic enzyme 1, NADP(+)-dependent, cytosolic | 1.3 | 0.01 |
| Aqp7 | aquaporin 7 | 1.4 | 0.002 |

**Supplementary Table S3: Proteins in myoglobin deficient *vs.* wild-type mouse heart tissue**

| Proteins | Log2(FC) | -Log10(P) |
|---|---|---|
| Inositol monophosphatase 2 | 1.7 | 4.2 |
| Methyl-CpG-binding protein 2 | 1.4 | 4.0 |
| 1.5-anhydro-D-fructose reductase | 3.0 | 3.9 |
| Muscle-related coiled-coil protein | 1.4 | 3.9 |
| Inositol monophosphatase 1 | 1.4 | 3.7 |
| Serine/threonine-protein kinase TNNI3K | 3.2 | 3.7 |
| Very-long-chain enoyl-CoA reductase | 1.1 | 3.6 |
| PDZ and LIM domain protein 3 | 1.0 | 3.6 |
| Desmoglein-2 | 1.1 | 3.5 |
| Very-long-chain (3R)-3-hydroxyacyl-CoA dehydratase 1 | 1.3 | 3.5 |
| Ribose-phosphate pyrophosphokinase 1 | 1.0 | 3.3 |
| Puromycin-sensitive aminopeptidase | 1.0 | 3.0 |
| Centromere protein V | 1.0 | 2.6 |
| Mitochondrial 10-formyltetrahydrofolate dehydrogenase | 2.3 | 2.6 |
| RNA-binding protein FUS | 1.0 | 2,6 |
| Pyruvate dehydrogenase kinase isozyme 4, mitochondrial | 2.1 | 2.3 |
| Putative RNA-binding protein Luc7-like 2 | 1.3 | 2.2 |
| 28S ribosomal protein S15, mitochondrial | 1.5 | 2.1 |
| Glutathione S-transferase theta-1 | 1.8 | 2.0 |
| Carboxylesterase 1D | 1.0 | 1.9 |
| Chloride intracellular channel protein 4 | 1.9 | 1.8 |
| Protein phosphatase methylesterase 1 | 1.3 | 1.6 |
| Integrin alpha-6 | 1.0 | 1.6 |
| Methylthioribose-1-phosphate isomerase | 1.1 | 1.6 |
| DCN1-like protein 2 | 1.2 | 1.4 |
| 4-aminobutyrate aminotransferase, mitochondrial | -3.1 | 1.3 |
| Heterogeneous nuclear ribonucleoproteins C1/C2 | -3.2 | 1.4 |
| Myoglobin | -9.3 | 1.4 |
| Ubiquitin-like modifier-activating enzyme 5 | -1.2 | 1.7 |
| 39S ribosomal protein L24. mitochondrial | -1.4 | 1.7 |
| CapZ-interacting protein | -1.3 | 1.8 |
| Phosphotriesterase-related protein | -2.4 | 1.9 |
| CDGSH iron-sulfur domain-containing protein 3, mitochondrial | -1.3 | 1.9 |
| Tetratricopeptide repeat protein 38 | -2.4 | 2.4 |
| Tripartite motif-containing protein 72 | -1.5 | 2.8 |
| Pre-B-cell leukemia transcription factor-interacting protein 1 | -2.7 | 2.9 |
| GTPase IMAP family member 4 | -1.3 | 3.0 |
| Vinexin | -1.9 | 3.5 |

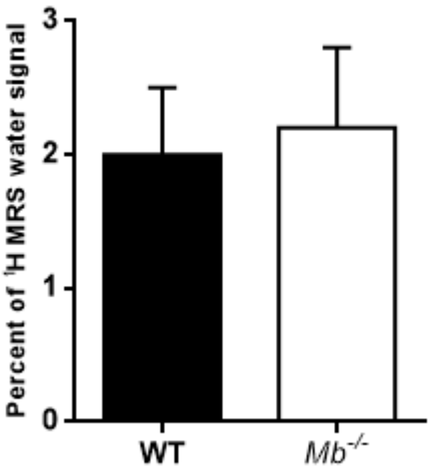


**Supplementary Fig. S1.** Quantitative analysis of the lipid content in young WT and *Mb*$^{-/-}$ mouse hearts (value as mean ± SD; *P* = 0.8, *n* = 5).

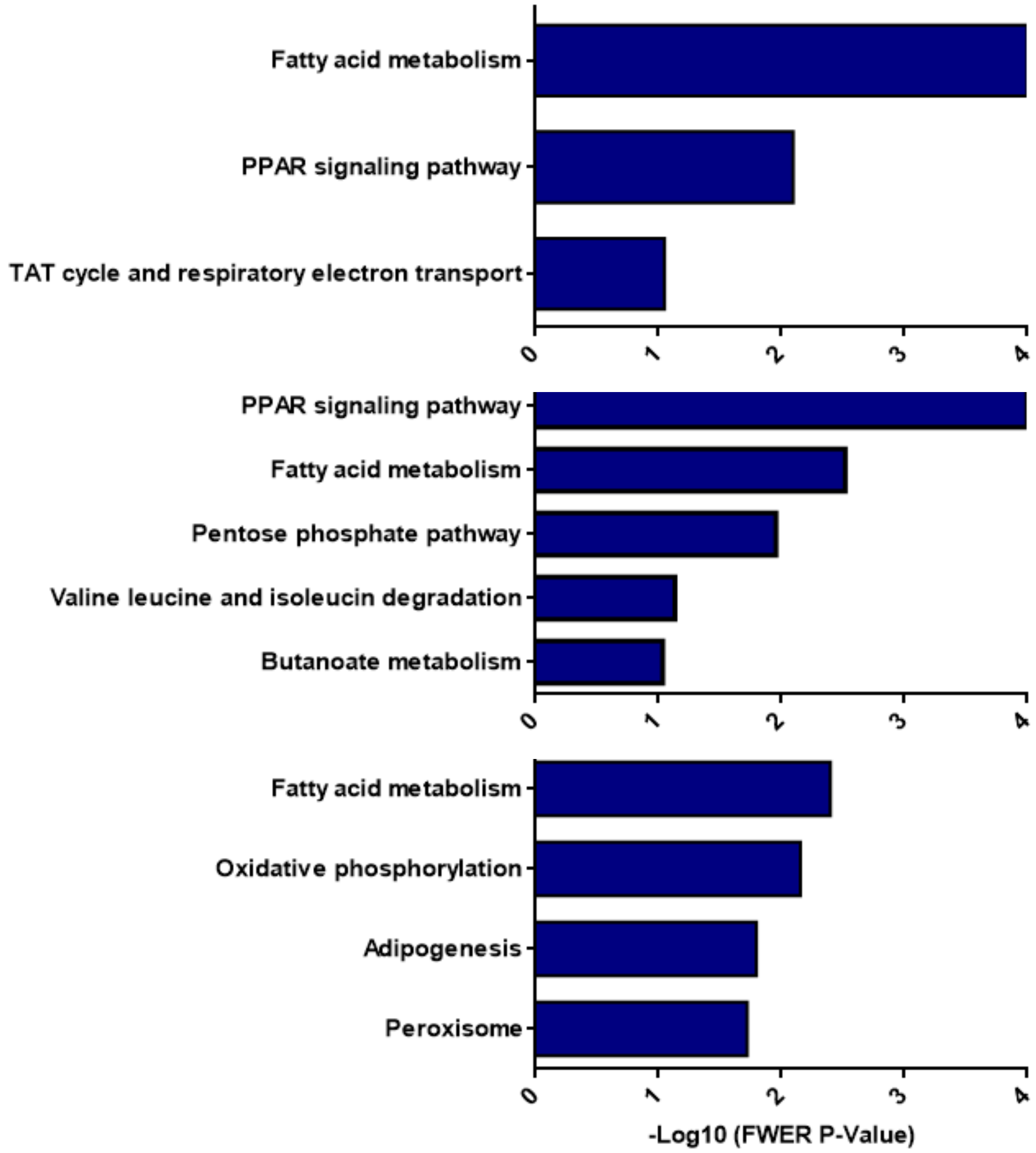


**Supplementary Fig. S2.** Functional categorization of dysregulated genes in the *Mb*$^{-/-}$ mouse hearts compared with WT mouse hearts. Upper panel: Reactome pathways, middle panel: KEGG pathways, lower panel: Hallmark pathways. Individual pathways are sorted by negative log [FWER *P*-value]. $n = 3$.
